# Ensemble Learning Based on Bagging and Hybrid Sampling for Food Safety Risk Prediction

**DOI:** 10.3390/foods15071176

**Published:** 2026-03-31

**Authors:** Dafang Li, Zhengyong Zhang, Qingchun Wu, Xin Chen

**Affiliations:** 1 School of Management Science and Engineering, Nanjing University of Finance and Economics, Nanjing 210023, China; zyzhang@nufe.edu.cn (Z.Z.); njuechx@163.com (X.C.); 2 Key Laboratory of Food Processing and Quality Control, Nanjing University of Finance and Economics, Nanjing 210023, China; 3 Humanities and Social Sciences Laboratory of Jiangsu Province, Food Safety and National Strategic Governance, Jiangnan University, Wuxi 214122, China; 4 Shenzhen Research Institute, Nanjing University of Aeronautics and Astronautics, Nanjing 210016, China; wqc520@nuaa.edu.cn

**Keywords:** food safety risk prediction, ensemble learning, bagging, hybrid sampling

## Abstract

Food safety sampling inspections are critical for risk prevention in complex supply chains, yet the extremely low frequency of high-risk samples poses substantial challenges for accurate risk prediction. To address the limitations of conventional machine learning models under severe class imbalance, this study proposes a unified Bagging–Stacking framework that integrates stacking ensembles, bagging, and SMOTE–Tomek hybrid resampling to enhance minority-class detection in food safety risk prediction. The stacking ensemble serves as the core of the framework, combining five tree-based base learners with Logistic Regression as the meta-learner to enhance classification robustness. Balanced bootstrap subsets generated through bagging and SMOTE–Tomek hybrid resampling further improve minority-class representation, while a probability-based threshold optimization mechanism is incorporated to refine high-risk classification. Experiments on real-world inspection data show that the proposed framework substantially improves high-risk recall while simultaneously increasing precision, yielding the highest F1 among all compared models. It also maintains a stable overall performance across varying test set proportions, demonstrating strong robustness and consistent generalization under varying evaluation conditions. SHAP analysis identifies storage conditions, production month, shelf life, package, and food category as key contributors to risk prediction, aligning with established mechanisms of food safety risk formation. Overall, the proposed framework provides accurate, robust, and interpretable support for food safety risk prediction, offering practical value for proactive risk prevention and more efficient regulatory resource allocation.

## 1. Introduction

Food safety is fundamental to public health and social stability, especially as modern food systems become increasingly globalized and complex [1]. Ensuring food safety requires effective identification and control of diverse hazards, including pathogenic microorganisms, heavy metals, pesticide and veterinary drug residues, as well as regulatory violations such as illegal additives and label fraud [2]. Many of these food safety risks typically exhibit low incidence rates but high severity, and failure to identify and intervene may lead to foodborne disease outbreaks and regional public health emergencies [3]. Globally, the consumption of unsafe food results in an estimated 600 million reported foodborne illnesses and 420,000 deaths each year, with 30% of foodborne deaths occurring among children under 5 [4].

In response to these challenges, government market regulatory authorities increasingly rely on large-scale monitoring and sampling programs to detect emerging risks. Yet manual inspection methods are plagued by prolonged cycles, high labor costs, and elevated error rates when handling large sample volumes [5], making machine learning a transformative solution to enhance inspection efficiency [6]. However, statistics released by China’s State Administration for Market Regulation (SAMR) show that unqualified samples constituted only 2.96% of all food sampling inspections conducted in 2024 (https://www.samr.gov.cn/spcjs/xxfb/art/2025/art_56ce6823a2194af4b2e652b425b316d9.html) (accessed on 5 January 2026), meaning that high-risk samples represent an extremely small proportion of the dataset [7]. This class imbalance often causes machine learning models to be dominated by majority classes during training, biasing predictions toward medium-risk or low-risk outcomes and resulting in inadequate recall of high-risk samples. Consequently, balancing the accurate identification of minority high-risk classes with the maintenance of overall model performance has become a central scientific challenge in intelligent food safety regulation [8].

Various techniques have been developed to mitigate class imbalance at the data, algorithm, or model level. Among data-level approaches, widely used methods include Random Undersampling (RUS), Synthetic Minority Oversampling Technique (SMOTE), and hybrid strategies such as SMOTE combined with Tomek Links (SMOTE–Tomek) [9,10,11,12]. SMOTE generates synthetic minority samples through interpolation, whereas Tomek Links identifies borderline instance pairs to remove noisy majority samples near the decision boundary. By integrating oversampling and undersampling, SMOTE–Tomek enhances minority-class representation while cleaning ambiguous majority samples, thereby improving classification performance [13,14]. However, SMOTE–Tomek still faces two inherent limitations in highly skewed food safety data: (i) when high-risk samples are extremely scarce, SMOTE can only interpolate within a very narrow neighborhood, resulting in limited diversity of synthetic samples; (ii) SMOTE–Tomek produces only a single balanced dataset, making the learned minority-class boundary sensitive to noise and local structures. To address these limitations, this study introduces bagging at the data level. Bagging, short for Bootstrap Aggregating, constructs multiple bootstrap-resampled subsets to reduce variance and enhance prediction stability. In imbalanced learning, bagging has been adopted to stabilize minority-class decision boundaries, and prior studies have explored its combination with sampling techniques, such as RSYNBagging and ADASYNBagging [15], to improve minority-class recognition. Nevertheless, few studies have integrated bagging with SMOTE–Tomek, particularly in the context of food safety risk prediction. Building on this gap, the study adopts a Bagging–SMOTE–Tomek hybrid resampling strategy in which multiple bootstrap subsets are generated, and each subset is independently balanced using SMOTE–Tomek. This design simultaneously provides inter-subset diversity and intra-subset class balance, forming a more robust foundation for subsequent model training.

Algorithm-level approaches for handling class imbalance mainly include class-weighting and output-thresholding techniques. Class-weighting directly modifies the learner by assigning higher penalties to minority-class errors [16]. Output thresholding, in contrast, adjusts the decision threshold used to convert probability estimates into class labels [17,18]. Although both approaches help reduce bias toward the majority class, thresholding offers a more practical and model-agnostic solution because it leaves the underlying learner unchanged and can be applied to any classifier that outputs probability scores. Therefore, this study focuses on threshold optimization.

Model-level approaches focus on enhancing the model’s ability to learn complex feature representations by optimizing model architectures. Traditional single models, including Bayesian Networks (BNs) [19,20], Support Vector Machines (SVMs) [21,22], Multi-Layer Perceptrons (MLPs), and Artificial Neural Networks (ANNs) [23,24], have been widely applied to classification and risk identification tasks in multi-source food safety monitoring. However, under extreme data imbalance, these models tend to be dominated by the majority class.

Ensemble learning, encompassing tree-based ensemble methods such as Random Forest (RF), Gradient Boosting (GB), Extreme Gradient Boosting (XGBoost), Light Gradient Boosting Machine (LightGBM), and Categorical Boosting (CatBoost), and heterogeneous stacking, plays a pivotal role in risk prediction tasks [25,26,27,28]. Among these, stacking ensemble learning offers notable advantages in handling high-dimensional nonlinear data and alleviating overfitting through the complementary integration of heterogeneous base models [29]. A representative study by Qin et al. demonstrated that combining hybrid sampling with stacking ensemble learning can effectively improve food safety sampling inspection classification performance [30].

**Figure 1:**
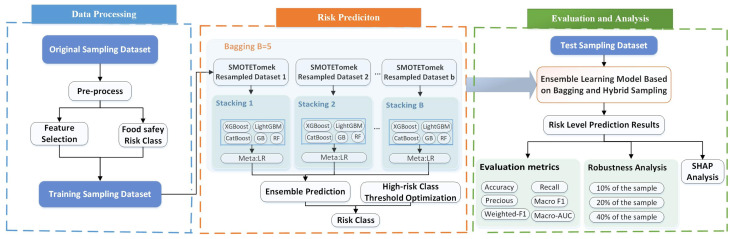
The unified Bagging–Stacking Framework for food safety risk prediction.

Although previous studies have explored sampling strategies, class-weighting schemes, and various ensemble learning techniques, existing approaches still struggle to simultaneously ensure model robustness, generalization, and effective high-risk sample identification in highly imbalanced real-world food safety inspection scenarios. To overcome these limitations, this study proposes a unified Bagging–Stacking framework (Figure 1) that advances food safety risk prediction through coordinated innovations at the data, model, and algorithm levels, which has not been systematically examined in prior work. At the data level, we design a Bagging–SMOTE Tomek hybrid resampling strategy to generate diverse and balanced bootstrap subsets. At the model level, we construct a heterogeneous stacking ensemble that combines five tree-based base learners with Logistic Regression as the meta-learner, enabling complementary model strengths to improve unqualified-sample detection accuracy. At the algorithm level, a probability-based threshold optimization mechanism was introduced to further strengthen high-risk class detection without modifying the underlying learners. Through the coordinated interaction of these three components, experimental results show that the proposed framework achieves substantially improved high-risk sample recall while maintaining stable overall classification performance—an advancement that cannot be achieved by any single component alone. This integrated design represents a meaningful methodological contribution to food safety risk prediction under real-world class imbalance.

## 2. Materials and Methods

### 2.1. Data Source and Pre-Processing

The food safety sampling inspection data utilized in this study were derived from the unqualified food samples inspection records of the National Municipal Market Supervision Administration in China during 2021–2025, covering all 32 provincial-level administrative regions of China. The sampling records encompass fields such as food category, declared manufacturer name and address, sampled unit name and address, sample name, package, production date, shelf life, and unqualified item. These records are characterized by heterogeneous origins, irregular text formats, and incomplete information fields. To clarify, in this study, an “unqualified sample” refers to any sample that fails at least one inspection item defined in the corresponding Chinese National Food Safety Standards. A sample is therefore classified as unqualified when any tested parameter, such as microbial indicators, heavy metals, nutritional components, or other physicochemical criteria, does not meet the regulatory limits. The specific unqualified item is recorded in the “unqualified item” field of the dataset.

To enhance data quality and ensure the reliability of subsequent modeling, the original sampling data underwent systematic cleaning and normalization, including: (1) standardizing text fields such as food categories and package, and uniformly classifying sampled online platforms (e.g., JD.com, Taobao.com) as “online store”, (2) imputing missing temporal data of production date and shelf life using the most frequently occurring values within the corresponding product batches, (3) standardizing Chinese text in unqualified item fields by removing special symbols and merging synonymous expressions to provide consistent input for risk class generation. Following these procedures, 792 valid samples were retained, covering 30 food categories.

### 2.2. Food Safety Risk

The hazard severity of unqualified items serves as an important basis for constructing risk prediction models in food safety sampling inspections. Recent studies have further demonstrated that different types of contaminants, pathogenic microorganisms, food additives, and quality-related indicators vary substantially in their associated health risks and exposure levels [31]. These differences in toxicological hazards and exposure levels constitute the scientific foundation of risk-level classification in China’s food safety risk assessment system [32]. Drawing on these regulatory practices and toxicological principles, this study categorises unqualified items into three risk classes—high, medium, and low—to construct the model’s risk-class classification system.

High-risk items primarily include heavy metal contamination, pathogenic microorganisms, residues of highly toxic pesticides or veterinary drugs, and illegal additives. Heavy metals such as lead, cadmium, mercury, and arsenic exhibit significant chronic toxicity, with dietary exposure and associated health risks repeatedly confirmed in monitoring and assessment studies across multiple regions of China [33]. Pathogenic bacteria such as Staphylococcus aureus and Salmonella can cause acute foodborne illnesses and are key targets for food safety regulation. Recent examples of foodborne illnesses include the 100 illnesses from Staphylococcus aureus exposure at the Coachella festival in California (https://www.foodsafetynews.com/2022/05/staph-blamed-for-foodborne-illness-outbreak-among-coachella-bus-drivers/) (accessed on 5 January 2026) and the outbreak of Salmonella in Finland that affected more than 700 people (https://www.foodsafetynews.com/2022/05/large-salmonella-outbreak-dominates-finnish-figures/) (accessed on 5 January 2026). Additionally, pesticide residues continue to be detected in certain foods, posing potential health risks [34].

Medium-risk items largely consist of excessive use of food additives and abnormal general microbial indicators. Food additives such as benzoic acid and sorbic acid may pose potential health risks to sensitive populations when used in excess [35]. General microbial indicators like total bacterial count and coliform bacteria do not directly cause severe foodborne illnesses. However, they reflect inadequate hygiene control during production and processing, which may increase risks of cross-contamination or spoilage [36].

Low-risk items are mainly associated with quality issues, such as nutritional content falling below standards, minor deviations in physicochemical indicators, and so on [37]. Although these issues typically do not directly endanger consumer health, they may compromise product quality or mislead consumers, thereby justifying their classification as low-risk.

**Figure 2:**
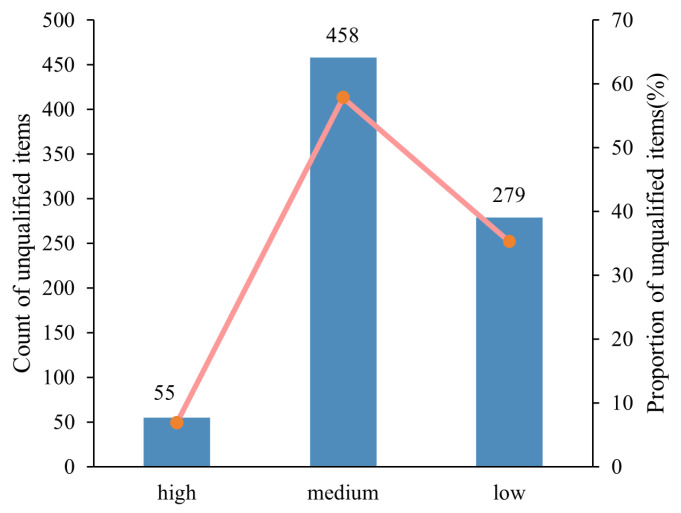
The number and proportion of unqualified items across different risk classes.

**Figure 3:**
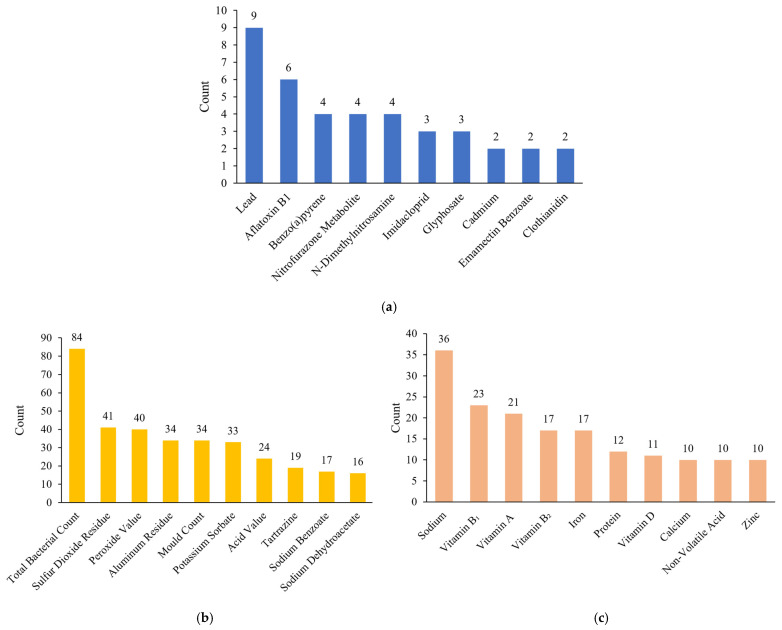
Counts of the top 10 unqualified items across the three risk classes. (**a**) High-risk items; (**b**) medium-risk items; (**c**) low-risk items.

Based on the above risk-class classification system, this study assigned risk classes to food safety unqualified inspection records and summarized the number and proportion of unqualified items across different risk classes (Figure 2 and Figure 3). Medium-risk samples accounted for the largest proportion at 57.83% (458 samples), mainly involving abnormalities in general microbial indicators (primarily total bacterial count and mould count), excessive use of food additives (e.g., sulfur dioxide residue, potassium sorbate), and lipid oxidation or deterioration (e.g., peroxide value, acid value), indicating the widespread occurrence of such issues in the sampling data. Low-risk samples represented 35.23% (279 samples), largely due to nutritional components failing to meet standards or falling below 80% of labelled values, particularly insufficient sodium, vitamins, iron, and calcium, which pose relatively low health risks. High-risk samples constituted only 5.68% (55 samples), mainly attributable to heavy metal contamination (primarily lead), biotoxins (e.g., aflatoxin B1), and highly toxic pesticide or veterinary drug residues (e.g., nitrofurazone metabolite, benzo(a)pyrene). Although the number of high-risk samples was limited, these hazards have direct implications for human health, carry substantial potential risks, and therefore warrant heightened regulatory attention.

Overall, the risk-class distribution reveals a pronounced class imbalance. High-risk samples constitute only a very small proportion yet carry substantial regulatory significance, imposing greater demands on the model’s ability to accurately identify them. Medium-risk samples represent the largest share and thus play a central role in determining overall classification performance. This imbalanced distribution highlights the need to incorporate resampling and ensemble learning strategies during model development to mitigate bias toward majority classes and to ensure more effective identification of high-risk samples.

### 2.3. Ensemble Learning Method Based on Bagging and Hybrid Sampling for Food Safety Risk Prediction

Traditional food safety risk prediction models often face substantial challenges when applied to highly imbalanced datasets. Owing to the extremely low proportion of high-risk samples in inspection data, single models and conventional stacking methods are prone to bias toward majority classes, resulting in markedly low recall for the high-risk class. In addition, model performance is highly sensitive to a single train–test split, making it challenging to achieve stable and reliable results in real-world regulatory contexts. To address these limitations, this study proposes a unified Bagging–Stacking framework, which integrates bagging, hybrid resampling, stacking ensembles, and probability-based threshold optimization. This integrated approach enhances the model’s ability to identify high-risk samples while improving robustness and overall classification stability.

Step 1: Bagging–SMOTE–Tomek hybrid resampling.

The original training sampling set is defined as

(1) $$D_{train}={\left\{(x_{i},y_{i})\right\}}_{i=1}^{N_{train}}$$

where $x_{i}$ is the feature vector of the i-th sample, $y_{i}$ denotes its risk class (0: low, 1: medium, 2: high). $D_{train}$ is obtained by stratified random sampling from the full dataset D to preserve the original class distribution. $N_{train}$ is the number of training sample sets.

In the b-th bagging iteration, SMOTE–Tomek resampling is applied to the training sample set to generate a balanced training subset:

(2) $$D_{train}^{\left(b\right)}=SMOTETomek(D_{train}),b=1,2,\ldots ,B$$

where B denotes the total number of bagging iterations in this study.

The SMOTE algorithm generates synthetic samples for the minority class through linear interpolation:

(3) $$x_{\mathrm{new}}=x_{i}+\lambda (x_{j}-x_{i}),\;\lambda \sim U(0,1)$$

where $x_{i}$ and $x_{j}$ are neighboring high-risk class samples, and λ is a random interpolation coefficient drawn from a uniform distribution. Tomek Links remove noisy majority class samples at the class boundary. If a sample pair $\left(x_{i},x_{j}\right)$ satisfies

(4) $$d(x_{i},x_{j})=\min\{d(x_{i},x_{k}),d(x_{j},x_{k})\}\;y_{i}\neq y_{j}$$

where $d(x_{i},x_{j})$ denotes the Euclidean distance.

This process ensures that each bagging round learns from a balanced and cleaner training subset, fundamentally improving the model’s sensitivity to the high-risk class.

Step 2: Training of stacking base learners.

On each resampled dataset $D_{train}^{b}$, a simple k-fold cross-validation (k=3) is employed to generate meta-features for the second-level meta-learner. 3-folds are used to balance computational cost and the stability of out-of-fold meta-feature estimation. Let $D_{train}^{b}$ be partitioned into 3 disjoint folds:

(5) $$D_{\mathrm{train}}^{\left(b\right)}=\overset{3}{\underset{k=1}{\cup }}F_{k}^{\left(b\right)},\;F_{i}^{\left(b\right)}\cap F_{j}^{\left(b\right)}=\emptyset \;(i\neq j)$$

where $F_{k}^{\left(b\right)}$ denotes the k-th fold of the training data in the b-th bagging round, with each fold serving as a validation set in turn.

For each base learning algorithm, a model is trained on the data, excluding the *k*-th fold:

(6) $$h_{m,k}^{\left(b\right)}=L_{m}\left(D_{\mathrm{train}}^{\left(b\right)}\setminus F_{k}^{\left(b\right)}\right),\;m=1,\ldots ,M,\;k=1,2,3$$

where *L_m_* denotes the *m*-th base learning algorithm, $h_{m,k}^{\left(b\right)}$ represents the trained *m*-th base learner on the *k*-th fold in the *b*-th bagging round, and *M* is the number of base learners. The base learners selected in this study include RF, GB, XGBoost, CatBoost, and LightGBM based on their superior performance in preliminary experiments.

The trained model $h_{m,k}^{\left(b\right)}$ is then used to generate predictions for samples in the held-out fold $F_{k}^{\left(b\right)}$. Each base learner outputs a three-class probability vector:

(7) $$P_{m,k}^{\left(b\right)}(y\left|x\right.)=h_{m,k}^{\left(b\right)}(y\left|x\right.)={\left[P_{Low},P_{Medium},P_{High}\right]}^{T},\forall x\in F_{k}^{\left(b\right)}$$

where $P_{m,k}^{\left(b\right)}(y\left|x\right.)$ represents the probability output of the *m*-th base learner on the *k*-th fold in the *b*-th bagging round for the sample *x*, and $P_{Low}$, $P_{Medium}$, $P_{High}$ are the predicted probabilities for each risk class.

The out-of-fold predictions from all folds are concatenated to form the meta-feature matrix for the *m*-th base learner:

(8) $$Z_{m}^{\left(b\right)}=\overset{3}{\underset{k=1}{\cup }}\left\{P_{m,k}^{\left(b\right)}(y|x):x\in F_{k}^{\left(b\right)}\right\}$$

The outputs of all base learners are concatenated to form the input for the second-level meta-learner:

(9) $$Z^{\left(b\right)}=\left[Z_{1}^{\left(b\right)},Z_{2}^{\left(b\right)},\ldots ,Z_{M}^{\left(b\right)}\right]$$

where $Z^{\left(b\right)}$ is the meta-feature matrix for the b-th bagging round.

The meta-learner (Logistic Regression (LR)) outputs the final fused probability for the b-th stacking ensemble:

(10) $$P_{Stacking}^{\left(b\right)}(y\left|x\right.)=Logistic(Z^{\left(b\right)})$$

Step 3: Ensemble Prediction.

For the sample x, the B stacking models yield B probability outputs $\left\{P_{Stacking}^{\left(1\right)}(y\left|x\right.),P_{Stacking}^{\left(2\right)}(y\left|x\right.),\ldots ,P_{Stacking}^{\left(B\right)}(y\left|x\right.)\right\}$.

The final prediction probabilities for each class are obtained through soft voting, i.e., averaging the probability outputs of all B stacking ensembles:

(11) $$P_{final}(y=c\left|x\right.)=\frac{1}{B}\sum_{b=1}^{B}P_{Stacking}^{\left(b\right)}(y=c\left|x\right.),\;c\in \{Low,Medium,High\}$$

where $P_{final}(y=c\left|x\right.)$ is the ensemble-averaged probability that the sample x belongs to class c.

Step 4: High-risk class threshold optimization.

The conventional multi-class decision rule is

(12) $$\hat{y}=\arg\max_{c}P_{final}(y=c\left|x\right.)$$

where $\hat{y}$ denotes the predicted risk class for the sample x.

To further improve the recall of the high-risk class, an independent threshold τ is introduced. If

(13) $$P_{final}(y=High\left|x\right.)>\tau$$

The sample is directly classified as a high-risk class. The final decision rule becomes

(14) $$\overset{⌢}{y}=\left\{\begin{array}{ll} \mathrm{High}, & \mathrm{if}\;P_{\mathrm{final}}(y=\mathrm{High}\left|x\right.)>\tau \\ \arg\max_{c\in \{Low,Medium\}}P_{final}(y=c\left|x\right.) & \mathrm{otherwise} \end{array}\right.$$

## 3. Results and Discussion

### 3.1. Data Description

**Figure 4:**
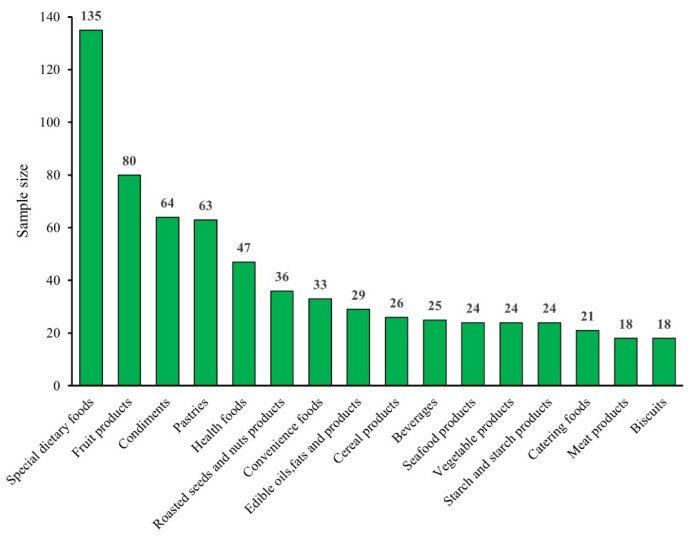
The number of unqualified samples of the food category.

Figure 4 shows the number of unqualified samples across the top 16 food categories. Among these, special dietary foods, fruit products, condiments, pastries, and health foods had the highest number of unqualified samples, at 135, 80, 64, 63, and 47, respectively.

### 3.2. Feature Selection

Feature engineering is a critical component in constructing food safety risk prediction models, as the quality of engineered features directly influences the model’s generalization capability and risk identification performance. Drawing on the feature characteristics of food safety regulatory scenarios, this study systematically derived features from original sampling inspection data and constructed a set of eight input variables, including food category, cross-province, sampling province, sampling site, package, production month, shelf life, and storage condition, as shown in Table 1. In addition, one output variable, risk class, was defined as the target for model prediction.

Given the pronounced seasonal patterns in food production, this study employed cyclical encoding for production months (Month-Sin and Month-Cos) to capture potential seasonal risk patterns. Previous research indicates that periodic encoding effectively enhances a model’s ability to represent seasonal and cyclical characteristics, making it suitable for time-dependent tasks such as food risk assessment [38,39]. Shelf life was categorized into three intervals, short (less than 6 months), medium (6–18 months), and long (greater than 18 months) to reflect differences in storage stability and spoilage risk across food products. Additionally, this study constructed cross-province circulation features by categorizing production–distribution relationships into four groups: local production, cross-city production, cross-province production, and unknown. This design enables the model to capture potential cumulative risk effects arising from cross-regional food supply chain movements. Existing literature indicates that longer cross-regional distribution chains increase regulatory challenges and risk exposure probabilities [40]. Sampling province information was cleaned, grouped, and encoded using one-hot encoding to capture regional variations in food safety. Categorical variables such as food category, package, sampling site, and storage condition were likewise encoded using one-hot encoding. These variables have been shown in prior food safety studies to be strongly associated with microbial contamination, additive usage patterns, and quality deviations [41]. The dataset used in this study encompassed 30 distinct food categories. To mitigate redundant feature interference, zero-variance filtering was applied to all variables to ensure that the input features retained effective information content [42]. After the above processing, a high-dimensional feature matrix suitable for machine learning modeling was ultimately constructed, laying the foundation for subsequent model training.

### 3.3. Optimization of Stacking Ensemble Architecture

#### 3.3.1. Implementation Details

Unlike bagging and boosting, a stacking ensemble employs a two-layer architecture that enables high-class integration of multiple heterogeneous data. In the first layer, multiple heterogeneous base learners are trained on the original training data, and their predictions serve as inputs for the meta-learner in the second layer, which learns how to optimally fuse the outputs [43]. This approach preserves the base learners’ ability to represent different feature spaces while leveraging the meta-learner for effective integration [44].

To select appropriate base learners for the first layer, this study first conducted systematic preliminary experiments on a variety of single classifiers, including RF, GB, XGBoost, CatBoost, LightGBM, MLP, KNN, and SVM. The dataset was randomly split into training and test sets using an 80:20 ratio, with stratified sampling employed to ensure consistent class distribution. The parameter search ranges for all single classifiers are detailed in Table 2. To avoid data leakage, all feature engineering operations (including one-hot encoding, data cleaning, etc.) were completed before dataset splitting, but no sampling algorithms were applied to address class imbalance.

Based on the screening results of the above preliminary experiments, the stacking ensemble architecture was constructed. Subsequently, this study further introduced a bagging strategy to optimize the stacking ensembles, aiming to enhance their stability and the ability to identify minority classes. The complete Bagging–Stacking framework follows the four-step procedure described in Section 2.3.

First, for each bagging iteration b=1,2,…,B, SMOTE–Tomek is applied to the original imbalanced training set, generating a class-balanced subset $D_{b}$, where B is set to 5 in this study. Subsequently, a complete stacking model $M_{b}$ is independently trained on each balanced subset, following the stacking ensemble learning procedure detailed in Equations (5)–(10). This process produces an ensemble of B diverse stacking learners $\left\{M_{1},M_{2,\ldots ,}M_{B}\right\}$. During prediction, for a given sample x, the probability outputs of all B stacking models are aggregated via soft voting as described in Equation (11). To further enhance the identification of high-risk samples, a threshold optimization mechanism is applied to the ensemble-averaged high-risk probability. Samples with high-risk probability exceeding a predefined threshold τ are classified as high-risk, regardless of the soft voting outcome. This threshold adjustment strengthens the model’s sensitivity to the minority high-risk class while maintaining overall classification stability.

To ensure the reliability of the results, all experiments in this study were conducted using Python 3.9.13 in Spyder IDE (version 5.2.2) on a notebook computer equipped with an Intel Core i7-14650HX processor (2.20 GHz), manufactured by Intel Corporation, headquartered in Santa Clara, CA, USA.

#### 3.3.2. Evaluation Metrics

To address the class imbalance, model performance was evaluated using multiple metrics, including accuracy, precision, recall, macro-F1, weighted-F1, and macro-AUC. Accuracy represents the proportion of correctly predicted samples across all risk classes relative to the total sample size, as defined in Equation (15). While accuracy provides an intuitive overview of overall predictive performance under balanced class distributions, it is susceptible to domination by the majority class in imbalanced datasets, potentially masking the model’s capability to identify high-risk classes. Recall for class c measures the model’s ability to correctly identify all instances belonging to a class c, as shown in Equation (16). In the context of food safety prediction, the recall rate of the high-risk class is particularly crucial, as it directly reflects the model’s capacity to detect potential food safety hazards. Precision for class c quantifies the proportion of samples predicted as class c that truly belong to that class, as defined in Equation (17). F1 for class c provides a comprehensive evaluation of the model’s performance in detecting samples from that class by calculating the harmonic mean of precision and recall, as presented in Equation (18). Macro-F1 represents the arithmetic average of F1 across all classes, while weighted-F1 accounts for the sample size of each class, as detailed in Equations (19) and (20), respectively. AUC quantifies the trade-off between the False Positive Rate (FPR) and True Positive Rate (TPR) along the ROC curve. In this study, the One-vs-Rest strategy was employed to calculate AUC values for each class, as shown in Equation (21), with the arithmetic mean of these values taken as the macro-AUC, as illustrated in Equation (22).

(15) $$\mathrm{Accuracy}=\frac{\sum_{c\in \{Low,Medium,High\}}TP_{c}}{N}$$

where $TP_{c}$ denotes the number of samples whose true class is c and are correctly predicted as class c. N represents the total number of samples in the dataset.

(16) Recallc=TPcTPc+FNc, c∈{Low,Medium,High}

where $FN_{c}$ represents the number of samples that truly belong to class c but are incorrectly predicted as other classes.

(17) $$\mathrm{Pr}{\mathrm{ecion}}_{c}=\frac{TP_{c}}{TP_{c}+FP_{c}},\;c\in \{Low,Medium,High\}$$

where $FP_{c}$ is the number of samples that do not belong to the class c but are incorrectly predicted as the class c.

(18) $$F1_{c}=2\times \frac{{\mathrm{Precision}}_{c}\times {\mathrm{Recall}}_{c}}{{\mathrm{Precision}}_{c}+{\mathrm{Recall}}_{c}},\;c\in \{Low,Medium,High\}$$

where ${\mathrm{Precision}}_{c}$ and ${\mathrm{Recall}}_{c}$ refer to the precision and recall of the class c, respectively.

(19) $$\mathrm{Macro-}F1=\frac{1}{3}\sum_{c\in \{Low,Medium,High\}}F1_{c}$$

(20) $$\mathrm{Weighted-}F1=\sum_{c\in \{Low,Medium,High\}}w_{c}F1_{c},w_{c}=\frac{n_{c}}{N}$$

where $n_{c}$ denotes the number of samples belonging to class *c*. *N* is the total number of samples, and *w*_*c*_ is the proportion of the class *c* in the dataset.

(21) $$\begin{array}{l} {\mathrm{AUC}}_{c}=\int_{0}^{1}{\mathrm{TPR}}_{c}({\mathrm{FPR}}_{c}^{-1}(x))d(x)\\ {\mathrm{TPR}}_{c}=\frac{TP_{c}}{TP_{c}+FN_{c}},{\mathrm{FPR}}_{c}=\frac{FP_{c}}{FP_{c}+TN_{c}},c\in \{Low,Medium,High\} \end{array}$$

where ${\mathrm{TPR}}_{c}$ and ${\mathrm{FPR}}_{c}$ are the TPR and FPR for class c under a One-vs-Rest scheme. $TP_{c}$, $FP_{c}$, $FN_{c}$ and $TN_{c}$ represent the confusion matrix components for class c.

(22) $$\mathrm{Macro-AUC}=\frac{1}{3}\sum_{c\in \{Low,Medium,High\}}{\mathrm{AUC}}_{c}$$

where AUC_*c*_ is the area under the ROC curve for class *c*, computed using a ne vs Rest strategy.

Table 3 shows the performance of each classifier under the same conditions. To align with the goals of food safety risk prediction, this study adopts Recall for the high-risk class (Recall_High_) and Macro-F1 as the core evaluation metrics. Recall_High_ is the most crucial, directly quantifying the model’s capacity to detect high-risk foods, while Macro-F1 ensures a balanced evaluation across all classes. Weighted-F1 serves as a secondary indicator of overall model stability.

As shown in Table 3, RF exhibited the most prominent performance in terms of recall for the high-risk class (0.73), substantially outperforming the other models, while also achieving a relatively high Macro-F1 (0.74), indicating its distinct advantage in identifying high-risk samples. GB and XGBoost demonstrated excellent performance in both Macro-F1 (0.75–0.79) and Weighted-F1 (0.83–0.85), reflecting their capability to maintain class balance while delivering strong overall performance. Although CatBoost and LightGBM achieved slightly lower Recall_High_ compared to the top three models, their Macro-F1 (0.71–0.75) and Weighted-F1 (0.78–0.82) remained stable, exhibiting satisfactory comprehensive predictive ability.

In contrast, while MLP achieved acceptable performance in Weighted-F1 (0.83), its Recall_High_ was merely 0.36, which fails to meet the requirements of food safety prediction for high-risk sample identification. KNN and SVM exhibited relatively low performance in both Macro-F1 and Recall_High_, demonstrating suboptimal overall performance and minority class recognition capability.

Considering the models’ high-risk identification ability, class balance, and overall performance, RF, GB, XGBoost, CatBoost, and LightGBM were selected as the base learners for the first layer of the stacking ensemble. LR was chosen as the meta learner to fuse base learner outputs, leveraging complementary strengths while mitigating overfitting risks [45,46].

#### 3.3.3. Threshold Selection

In multi-class food safety risk prediction, high-risk classes are the primary focus of regulatory authorities. Although the default decision rule assigns samples to the class with the highest predicted probability, this approach may fail to maximize the recall rate for high-risk classes, particularly in cases of class imbalance. To address this issue, we introduce an independent decision threshold τ for the high-risk class probabilities $P(y=High\left|x\right.)$. As long as τ is met $P(y=High\left|x\right.)>\tau$, the sample is classified as a high-risk class regardless of the relative probabilities of the other classes.

**Figure 5:**
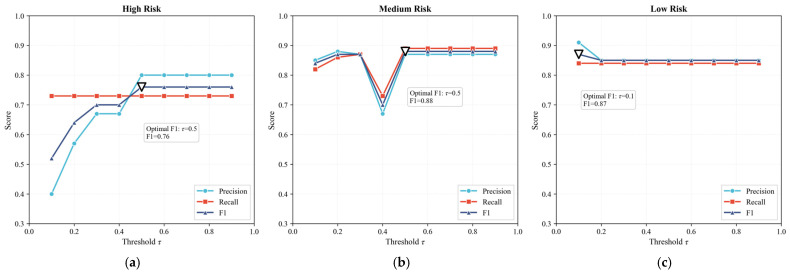
Threshold optimization for risk classification. (**a**) Threshold–performance curves for high-risk; (**b**) threshold–performance curves for medium-risk; (**c**) threshold–performance curves for low-risk.

**Figure 6:**
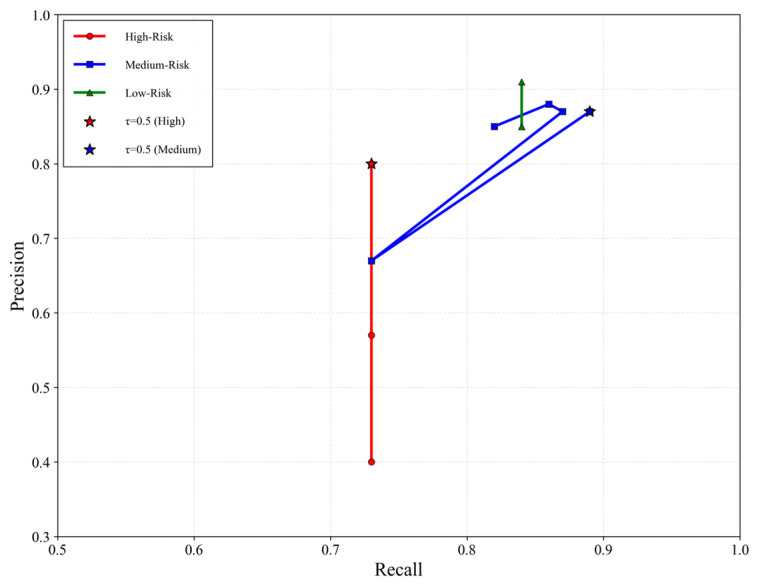
Precision–recall curves for three risk classes.

To rigorously justify the selection of τ, we conducted a systematic threshold sensitivity analysis. Specifically, we evaluated a series of discrete thresholds τ ranging from 0.1 to 0.9 with an increment of 0.1. For each threshold, the precision, recall, and F1 of all three risk classes were computed. This process generated a complete threshold–performance curve listed in Figure 5, enabling a systematic examination of how different decision thresholds affect model behavior. In addition, we constructed precision–recall curves (PR curve) for all classes (Figure 6) to further characterize the trade-off between precision and recall under varying thresholds. The PR curves provide a complementary global perspective by illustrating how precision improves as recall is maintained, thereby validating the threshold-dependent patterns observed in Figure 5.

Figure 5 showed that for the high-risk class, recall remains constant at 0.73 across all thresholds, while precision increases with the threshold and stabilizes at 0.80 for τ≥0.5. The PR curve in Figure 6 exhibits the same trend, confirming that this threshold achieves the most favorable precision–recall balance. The threshold τ=0.5 is the first threshold that attains the highest F1. For the medium-risk class, both the threshold–performance curves and the PR curve indicate that τ=0.5 provides the best combination of precision and recall, where the F1 stabilizes at 0.88. For the low-risk class, the F1 is slightly higher at τ=0.1 (0.87) and then stabilizes at 0.85 for τ≥0.2, and the PR curve shows minimal sensitivity to threshold changes, indicating that low-risk classification is inherently stable.

Based on these results, this study adopts τ=0.5 as the optimal threshold for high-risk classification, as it is the minimal threshold that attains the highest F1 for the high-risk class, aligns with the optimal operating range for the medium-risk class, and corresponds to the point where the PR curves enter a stable high-precision region.

#### 3.3.4. Performance Comparison

To validate the effectiveness of the proposed unified Bagging–Stacking framework, a comprehensive comparison was conducted with five well-performing single classifiers, namely RF, XGBoost, GB, CatBoost, and LightGBM. As shown in Table 4, the unified Bagging–Stacking framework achieved excellent accuracy (0.86), Weighted-F1 (0.86), and Macro-F1 (0.86), representing improvements of 1% to 10% in accuracy, 1% to 10% in Weighted-F1, and 5–12% in Macro-F1. These improvements can be regarded as moderate to substantial, particularly for Macro-F1, which is critical for imbalanced multi-class risk classification. Taken together, these results demonstrate the advantage of ensemble learning in complex risk scenarios, where integrated algorithms consistently outperform conventional single classifiers for food inspection data.

**Figure 7:**
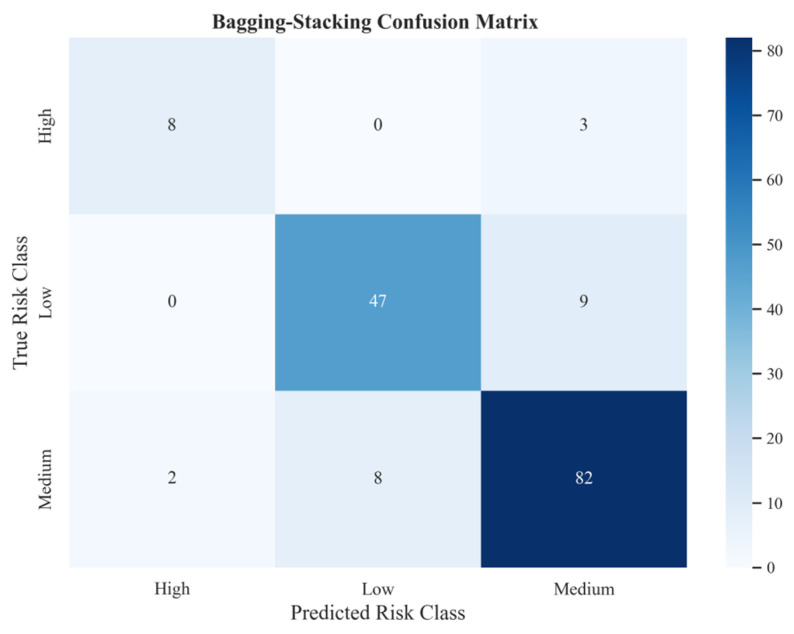
Confusion matrix of the unified Bagging–Stacking framework.

The advantage of the unified Bagging–Stacking framework was even more pronounced for the high-risk class. It achieved a high-risk recall of 0.73, matching the performance of the best-performing RF model (0.73) and substantially exceeding that of the other single models (0.45–0.55). In terms of F1 for the high-risk class, the unified Bagging–Stacking framework attained 0.76, notably outperforming all single models (0.52–0.71). Furthermore, its AUC reached 0.92, matching CatBoost at the optimal level. It is worth noting that although RF achieved the same high-risk recall as the unified Bagging–Stacking framework, its precision (0.67) and F1 (0.70) were both lower. This finding was further validated by the confusion matrix presented in Figure 7. Among the 11 high-risk samples, the unified Bagging–Stacking framework correctly identified 8 (73%), with the remaining 3 misclassified as medium-risk and none misclassified as low-risk. Although the number of high-risk samples was limited, this result still demonstrates the model’s satisfactory class discrimination capability for the high-risk class. In contrast, most single classifiers not only exhibited lower recall for the high-risk class but were also more prone to misclassifying high-risk samples as low-risk.

For the medium- and low-risk classes, the unified Bagging–Stacking framework maintained consistently strong performance. In the medium-risk class, it achieved a precision of 0.87 and a recall of 0.89, performing comparably to GB and slightly below XGBoost (0.92). Its F1 of 0.88 was close to GB (0.89) and higher than most single models. In the low-risk class, the model reached a precision of 0.85, a recall of 0.84, and an F1 of 0.85, all representing the best overall performance. These results demonstrate that the unified Bagging–Stacking framework enhances high-risk detection without compromising performance on the medium- and low-risk classes. The confusion matrix further shows that it correctly identified 47 low-risk samples, misclassifying only 9 as medium-risk and exhibiting no severe cross-level errors.

In summary, the unified Bagging–Stacking framework effectively balances the dual objectives of avoiding missed high-risk detections and ensuring stable identification of medium- and low-risk classes in highly imbalanced food safety sampling scenarios, offering a robust and reliable pathway for intelligent food safety supervision.

#### 3.3.5. Robustness Analysis Under Different Test Set Ratios

**Figure 8:**
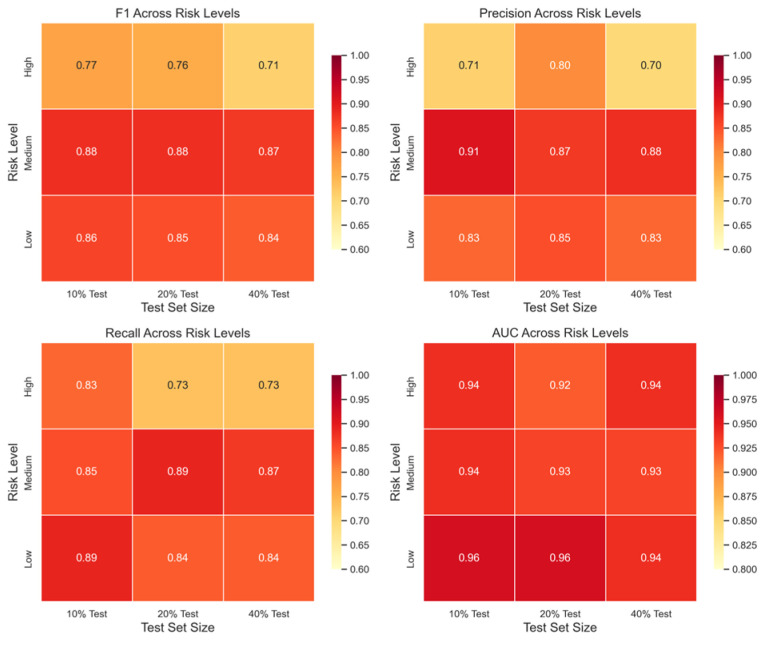
Heatmap of F1, precision, recall, and AUC across risk classes.

To assess the stability of the proposed unified Bagging–Stacking framework under varying data partitioning conditions, systematic evaluations were conducted using test set proportions of 10%, 20%, and 40%. Model performance across the three risk classes was examined using F1, precision, recall, and AUC, with the corresponding heatmaps presented in Figure 8.

For F1, the high-, medium-, and low-risk classes all maintained relatively stable performance across different test set proportions, within the ranges of 0.71–0.77, 0.87–0.88, and 0.84–0.86, respectively. These results indicate that the model preserves consistent classification quality across all three risk levels, even as the test set proportion increases.

For precision, the medium- and low-risk classes again showed stable and high performance, consistently remaining above 0.87 and 0.83, respectively, suggesting that the model’s positive predictive ability for the majority classes is largely unaffected by data partitioning. Although the high-risk class exhibited variability (0.71, 0.80, 0.70), its precision consistently stayed above 0.70, demonstrating that the model maintains a meaningful level of accuracy in identifying high-risk samples.

For recall, high-risk declined from 0.83 at the 10% test set to 0.73 at both the 20% and 40% settings, showing that the model continues to capture a meaningful proportion of high-risk instances even when fewer training samples are available. Meanwhile, recall for the medium- and low-risk classes remained consistently high (0.84–0.89), demonstrating robust performance for both classes.

Finally, the AUC heatmap shows that the model maintained consistently high AUC values (0.92–0.96) across all three risk classes, with only minor variation across test-set proportions. This indicates strong stability in probability-level risk ranking, even when the training–testing split changes substantially.

### 3.4. SHAP Analysis

**Figure 9:**
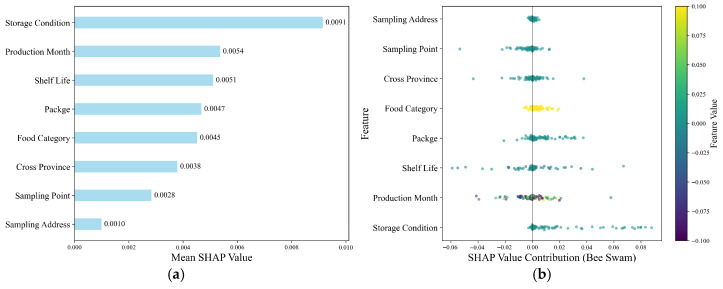
SHAP visualization. (**a**) Feature importance SHAP plot; (**b**) SHAP value contribution (bee swarm) plot.

To further interpret the decision-making mechanism of the unified Bagging–Stacking framework, SHAP (SHapley Additive exPlanations) analysis was applied (Figure 9). In panel (a), the X-axis shows mean absolute SHAP values, reflecting feature importance magnitude, while the Y-axis lists input features. Panel (b) illustrates the distribution of SHAP values across individual samples, indicating both contribution direction (positive or negative) and intensity.

Among all features, storage condition exhibited the highest mean SHAP value of 0.0091, followed by production month at 0.0054 and shelf life at 0.005, forming the three most significant features. Panel (b) shows that poor storage conditions (e.g., inadequate temperature control) generally correspond to positive SHAP values, thereby significantly increasing the probability of samples being classified as high risk. Meanwhile, the SHAP contribution of production month reflects seasonal risk, with samples produced during high-temperature periods more likely to be classified as high risk. Similarly, shorter shelf-life products show positive contributions, underscoring the combined importance of storage condition, seasonality, and product freshness in food safety risk determination. These findings are consistent with insights highlighting the critical role of storage, spoilage, and shelf-life in shaping food safety outcomes [47].

Package (0.0047) and food category (0.0045) also contributed notably to model predictions. Panel (b) indicated that unpackaged products generally correspond to positive SHAP values, indicating elevated risk classes [41]. The broad distribution of SHAP values across food categories suggests substantial heterogeneity in risk among product types, with the model effectively capturing structural relationships between category features and food safety risk.

Overall, the SHAP analysis demonstrates that the unified Bagging–Stacking framework possesses strong interpretability, reasonably reflecting the key factors underlying food safety risk formation.

### 3.5. Regulatory Implications

The proposed unified Bagging–Stacking framework demonstrates substantial advantages in addressing the challenges posed by highly imbalanced food safety inspection data. By incorporating a bagging mechanism into the stacking architecture, the framework effectively reduces the instability associated with single-split training and significantly enhances the identification of high-risk samples. Compared with traditional single classifiers, the unified Bagging–Stacking framework leverages the complementary strengths of multiple base learners, enabling it to capture subtle and heterogeneous risk signals embedded in noisy, real-world inspection data. This improved capability provides a quantitative foundation for designing more precise and adaptive sampling strategies.

From a regulatory perspective, the enhanced risk-recognition performance of the ensemble model supports the development of more targeted and resource-efficient inspection plans. Regulatory agencies can prioritize sampling for the high-risk class to optimize the use of limited inspection resources. When combined with the model’s probabilistic outputs and threshold optimization, these sampling priorities can be dynamically adjusted in response to seasonal patterns, emerging hazards, or policy needs. The optimized threshold (τ=0.5), identified in Section 3.3.3 as the first point attaining the highest F1, provides a practical decision boundary for regulatory use. Building on this optimized threshold, regulatory agencies can flexibly adjust the decision boundary according to inspection priorities. Lowering the threshold increases the model’s sensitivity, enabling inspectors to capture a broader range of potentially risky samples during high-risk periods (e.g., summer seasons or major events). Conversely, raising the threshold allows regulators to concentrate limited inspection resources on samples with substantially higher predicted unqualified probabilities, which is particularly valuable under resource-constrained conditions. This flexible thresholding mechanism enables a shift from static, uniform sampling to adaptive, risk-oriented inspection strategies.

The results of the SHAP analysis provide actionable insights for frontline food safety inspectors. The highest SHAP score for storage conditions suggests that food business operators with inadequate storage infrastructure, weak cold-chain management, or unstable temperature control should be flagged for more frequent sampling or targeted follow-up inspections. Similarly, food products manufactured during high-temperature seasons or those approaching the end of their shelf life are more prone to safety risks, indicating that regulatory authorities may need to increase the frequency of random inspections during the summer. The elevated SHAP values for unpackaged or minimally packaged foods further imply that these products are more susceptible to contamination during distribution and therefore require stricter on-site inspections. By integrating these risk factors into routine inspection workflows, frontline inspectors can allocate limited regulatory resources more efficiently and reduce the likelihood of overlooking high-risk products.

## 4. Conclusions

This study proposes a unified Bagging–Stacking framework that integrates multiple stacking ensembles and a Bagging–SMOTE–Tomek hybrid resampling strategy to address the pronounced class imbalance in food safety inspection data. Experimental results demonstrate that the proposed framework achieves substantially higher recall and more stable identification performance for the high-risk class compared with single-model baselines. Robustness analyses across varying test set proportions further reveal consistent trends in F1, precision, recall, and AUC for high-, medium-, and low-risk classes, indicating strong generalization capability under different data-splitting conditions. SHAP analysis also identifies the most influential risk factors. Overall, the unified Bagging–Stacking framework provides accurate, robust, and interpretable risk-prediction support for food safety regulation, enhancing the efficiency of regulatory resource allocation and strengthening the foresight of risk prevention and control.

Despite these strengths, the study has some limitations. The dataset originates solely from China’s SAMR system, with a relatively small sample size, and the severity of class imbalance varies across regions (e.g., some areas contain very few low-risk samples). Such geographical specificity and heterogeneous class distributions may limit the model’s generalisability and its applicability to other food safety systems, such as the EU’s RASFF. In addition, the absence of external information, such as supply-chain logistics data or enterprise production records, may also limit its ability to predict potential or hidden risks. Furthermore, the current dataset does not support identifying which specific food categories or packaging forms are most associated with high-risk hazards. Future work may incorporate multi-year or multi-regional datasets to evaluate cross-system applicability, integrate real-time data or supply-chain data to enhance risk prediction capability, explore alternative ensemble architectures to further improve predictive performance, and leverage more detailed product- and packaging-level information to better examine their associations with high-risk hazards. Additionally, combining SHAP analysis with intelligent inspector systems could provide more targeted regulatory strategies, while emerging technologies like IoT and multimodal AI also offer promising opportunities for more comprehensive and proactive food safety risk monitoring.

## Figures and Tables

**Table 1:** Feature attributes and values.

Feature	Feature Attributes and Values
Food category	Special dietary foods = 1, Fruit products = 2, Condiments = 3, Health foods = 4, Roasted seeds and nuts products = 5, Convenience foods = 6, etc.
Cross-province	Local production = 1, Cross-city production = 2, Cross-province production = 3, Unknown = 4
Sampling site	General store = 1, Grocery store = 2, Supermarket = 3, Restaurant = 4, Online store = 5, Farmers’ market = 6
Sampling province	One-hot encoding
Package	Unpackaged = 1, Prepackaged = 2
Production month	Cyclical encoding
Shelf life	Short Shelf life = 1, Medium Shelf life = 2, Long Shelf life = 3
Storage condition	Ambient Temperature Storage = 1, Refrigerated storage = 2, Frozen storage = 3
Risk class	Low = 0, Medium = 1, High = 2

**Table 2:** Classifier and parameter settings.

Classifier	Parameters	Parameters Values
XGBoost	n_estimators	150
	max_depth	6
	learning_rate	0.05
	min_child_weight	1
	subsample	0.8
	colsample_bytree	0.8
	random_state	42
	verbosity	0
	eval_metric	mlogloss
	objective	multi:softprob
RF	n_estimators	150
	max_depth	6
	min_samples_split	2
	min_samples_leaf	1
	class_weight	balanced
	random_state	42
	n_jobs	−1
LGBM	n_estimators	150
	max_depth	6
	learning_rate	0.1
	num_leaves	31
	min_child_samples	20
	subsample	0.8
	colsample_bytree	0.8
	random_state	42
	verbose	−1
GB	n_estimators	150
	max_depth	5
	learning_rate	0.1
	random_state	42
Catboost	iterations	150
	depth	6
	learning_rate	0.1
	l2_leaf_reg	3
	random_state	42
	verbose	FALSE
SVM	kernel	rbf
	C	1
	gamma	scale
	probability	TRUE
	random_state	42
	class_weight	balanced
KNN	n_neighbors	5
	weights	uniform
	algorithm	auto
MLP	hidden_layer_sizes	(100, 50)
	activation	relu
	solver	adam
	alpha	0.0001
	batch_size	auto
	learning_rate	constant
	learning_rate_init	0.001
	max_iter	500
	random_state	42

**Table 4:** Performance comparison of the unified Bagging–Stacking framework and baseline models.

Metric	RF	XGBoost	GB	Catboost	LighGBM	Bagging–Stacking
**High**						
Precision	0.67	1	0.50	1	0.71	0.80
Recall	0.73	0.55	0.55	0.45	0.45	0.73
F1	0.70	0.71	0.52	0.62	0.56	0.76
AUC	0.91	0.91	0.90	0.92	0.87	0.92
**Medium**						
Precision	0.82	0.83	0.89	0.82	0.80	0.87
Recall	0.77	0.92	0.89	0.89	0.84	0.89
F1	0.79	0.87	0.89	0.85	0.82	0.88
AUC	0.91	0.93	0.94	0.91	0.90	0.93
**Low**						
Precision	0.70	0.84	0.85	0.80	0.75	0.85
Recall	0.75	0.75	0.84	0.77	0.75	0.84
F1	0.72	0.79	0.85	0.78	0.75	0.85
AUC	0.92	0.93	0.96	0.93	0.93	0.96
Accuracy	0.76	0.84	0.85	0.82	0.78	0.86
Weight-F1	0.76	0.83	0.85	0.81	0.78	0.86
Macro-F1	0.74	0.79	0.75	0.75	0.71	0.83
Macro-AUC	0.92	0.93	0.94	0.92	0.90	0.94

**Table 3:** Performance comparison of different classifiers for risk prediction.

Classifier	Macro-F1	Weighted-F1	Recall_High_
RF	0.74	0.76	0.73
GB	0.75	0.85	0.55
XGBoost	0.79	0.83	0.55
CatBoost	0.75	0.81	0.45
LighGBM	0.71	0.78	0.45
MLP	0.70	0.83	0.36
KNN	0.67	0.78	0.27
SVM	0.50	0.62	0.36

## Data Availability

The original contributions presented in this study are included in the article. Further inquiries can be directed at the corresponding author.
